# Detection of N-Glycolyl GM3 Ganglioside in Neuroectodermal Tumors by Immunohistochemistry: An Attractive Vaccine Target for Aggressive Pediatric Cancer

**DOI:** 10.1155/2011/245181

**Published:** 2011-09-20

**Authors:** Alejandra M. Scursoni, Laura Galluzzo, Sandra Camarero, Jessica Lopez, Fabiana Lubieniecki, Claudia Sampor, Valeria I. Segatori, Mariano R. Gabri, Daniel F. Alonso, Guillermo Chantada, María Teresa G. de Dávila

**Affiliations:** ^1^Departament of Pathology, Pediatric Hospital “Prof. Dr. Juan P. Garrahan”, C1245AAM Buenos Aires, Argentina; ^2^Department of Hemato-Oncology, Pediatric Hospital “Prof. Dr. Juan P. Garrahan”, C1245AAM Buenos Aires, Argentina; ^3^Laboratory of Molecular Oncology, Quilmes National University, B1876BXD Buenos Aires, Argentina

## Abstract

The N-glycolylated ganglioside NeuGc-GM3 has been described in solid tumors such as breast carcinoma, nonsmall cell lung cancer, and melanoma, but is usually not detected in normal human cells. Our aim was to evaluate the presence of NeuGc-GM3 in pediatric neuroectodermal tumors by immunohistochemistry. Twenty-seven archival cases of neuroblastoma and Ewing sarcoma family of tumors (ESFT) were analyzed. Formalin-fixed, paraffin-embedded tumor samples were cut into 5 *μ*m sections. The monoclonal antibody 14F7, a mouse IgG1 that specifically recognizes NeuGc-GM3, and a peroxidase-labeled polymer conjugated to secondary antibodies were used. Presence of NeuGc-GM3 was evident in 23 of 27 cases (85%), with an average of about 70% of positive tumors cells. Immunoreactivity was moderate to intense in most tumors, showing a diffuse cytoplasmic and membranous staining, although cases of ESFT demonstrated a fine granular cytoplasmic pattern. No significant differences were observed between neuroblastoma with and without NMYC oncogene amplification, suggesting that expression of NeuGc-GM3 is preserved in more aggressive cancers. Until now, the expression of N-glycolylated gangliosides in pediatric neuroectodermal tumors has not been investigated. The present study evidenced the expression of NeuGc-GM3 in a high proportion of neuroectodermal tumors, suggesting its potential utility as a specific target of immunotherapy.

## 1. Introduction

Gangliosides are a broad family of glycosphingolipids found on the outer cell membrane, initially suggested as potential targets for cancer immunotherapy based on their higher abundance in tumors when compared with the matched normal tissues [1]. They are concentrated in the nervous tissues, particularly in gray matter and synaptic junctions, although they can also be detected in most cellular types in much smaller quantities. Gangliosides are involved in cell communication and also act as regulatory elements in the immune system and in cancer progression [2, 3]. 

Neuroblastoma, a neoplasm originating from neural crest cells, is the most common extracranial solid tumor of childhood. Although it can arise from any site along the sympathetic nervous system, primary tumors frequently have abdominal or thoracic location [4]. Aberrant expression of NMYC oncogene plays a central role in neuroblastoma tumorigenesis, and its amplification is a major indicator of high-risk cases [5]. Most patients achieve remission with chemotherapy and surgery, but eradication of minimal residual disease (MRD) remains the major challenge in improving prognosis in high-risk neuroblastoma [4, 5].

The expression of a variety of gangliosides has been described in detail in neuroblastoma for diagnostic purposes and more recently for therapeutic targeting, since disialoganglioside (GD2)-directed immunotherapy has been reported to improve survival in children with high-risk neuroblastoma [6]. Anti-GD2 monoclonal antibodies, as well as newer strategies such us immunocytokines and tumor vaccines, are promising approaches to eliminate resistant neuroblastoma cells [7].

The expression of N-glycolyl (NeuGc) gangliosides, and particularly the monosialoganglioside NeuGc-GM3, in neuroblastoma has not been reported to our knowledge. These gangliosides have recently received attention as a privileged target for cancer immunotherapy based on results in adult tumors [8]. Our group recently reported its expression in pediatric Wilms tumor [9]. Since this ganglioside is not detected in normal human cells, it may be considered as an interesting neoantigen for cancer immunotherapy [8, 10].

The monoclonal antibody racotumomab (formerly known as 1E10), an anti-idiotype vaccine that is able to induce a specific response against NeuGc-containing gangliosides, may be considered as an option for immunotherapy in these children. Racotumomab showed promising results in clinical trials in patients with advanced breast carcinoma [11, 12], melanoma, [13], and nonsmall cell lung cancer [14]. Furthermore, it was recently described that induction in cancer patients of anti-NeuGc-GM3 antibodies can cause tumor cell death by a complement-independent oncosis-like mechanism [15].

Based on this experience, we aimed to investigate the immunohistochemical expression of the N-glycolylated ganglioside NeuGc-GM3 in neuroblastoma in order to evaluate its potential as a target for immunotherapy. Cases of Ewing sarcoma family of tumors (ESFTs) were also studied, providing additional information in neuroectoderm-derived pediatric cancers.

## 2. Materials and Methods

### 2.1. Archival Cases

We retrospectively reviewed pathological specimens from 27 patients with a diagnosis of neuroblastoma or ESFT treated at the Garrahan Pediatric Hospital (Buenos Aires, Argentina). The median age of neuroblastoma patients was 22 months (range: 2 months to 11 years) at initial diagnosis. The most frequent primary site was abdominal, followed by thoracic, cervical, and axilar sites. The median age of ESFT patients was 13 years (range: 9 to 14 years), with all cases being diagnosed as primary extraosseous disease.

 Neuroblastoma tumors were immunohistochemically evaluated with the monoclonal antibody NB84 (Dako Cytomation, Carpinteria, CA, USA) raised to neuroblastoma cells, while cases of ESFT were examined using an anti-CD99 antibody (Dako Cytomation). Additional immunohistochemical markers were also assessed to confirm the diagnosis of neuroblastoma or establish a differential diagnosis with other small round blue cell tumors, including synaptophysin, neurofilaments, neuron-specific enolase, Myf4, terminal deoxynucleotide transferase, and vimentin. Patients with high-risk neuroblastoma were treated with current regimens based on evidence-based guidelines [16], thus they underwent surgical removal of the primary tumor after administration of 5 cycles of induction polychemotherapy. Children with low-risk neuroblastoma were treated with surgical resection only. Histological assessment and pathological staging were in accordance with the International Neuroblastoma Pathology Committe [17].

### 2.2. Fluorescent In Situ Hybridization (FISH)

NMYC amplification was detected by FISH with a molecular specific DNA probe for 2p24.1 (Vysis N-MYC, Spectrum Orange Probe, Abbot Molecular, Abbot Park, IL, USA) on histological sections of neuroblastoma tumors. The number of fluorescent signals was evaluated in 200 intact, nonoverlapping nuclei of tumor cells for each probe. Positive cases were considered when the signals were of the same size and intensity. Complementary, 1p deletion was checked in neuroblastoma using a DNA probe for 1p36 (locus D1Z2). ESFT cases were analyzed for the presence of *t*(11;22) translocation with a probe for the EWR1 locus at 22q12.

### 2.3. Immunohistochemistry

The antiganglioside monoclonal antibody 14F7 was provided by the Center of Molecular Immunology (La Habana, Cuba) and used at a final concentration of 20 *μ*g/mL. The 14F7 antibody is a mouse IgG1 that specifically recognizes the ganglioside NeuGc-GM3, as previously described [12]. Sections of 5 *μ*m from formalin-fixed, paraffin-embedded tumor samples were used. After reaction of primary antibodies, sections were incubated with a peroxidase-labeled polymer conjugated to secondary anti-mouse antibodies using the EnVision+ System-HRP(DAB) (DakoCytomation) and developed with 3,3′-diaminobenzidine as chromogen. Proper positive and negative controls were made in every staining battery. Sections from breast carcinoma were employed as positive controls of ganglioside detection [9]. We previously demonstrated that the routine technique did not extract antigenic carbohydrate determinants of gangliosides, thus allowing immunohistochemical detection in tumor sections [18]. Detection of Ki-67 protein, a nuclear marker of proliferating cells, was performed using the specific mouse monoclonal antibody MIB-1 (DakoCytomation) at a dilution of 1 : 50.

### 2.4. Immunohistochemical Evaluation

NeuGc-GM3 expression was semiquantitatively evaluated, and results were scored by three independent pathologists. In the rare event of divergent evaluation, a consensus was found by discussing the cases. We graded the intensity of the staining from 0 to 3 : 0 = no staining; 1+ = mild; 2+ = moderate; 3+ = intense. Tumors were classified as negative when no staining was observed or only less than 20% of cells were positive. In addition, neuroblastoma tumors were assessed with the immunoreactive score (IRS). The percentage of NeuGc-GM3 positive cells was quantified in 5 high-power fields (in average: 2,500 cells per case) and then scored in five grades: 0 = 0–19%; 1 = 20–39%; 2 = 40–59%; 3 = 60–79%; 4 = 80–100%. IRS was calculated for each specimen by multiplication of the staining intensity and the grade of positive cells, resulting in a score ranging from 0 to 12 as described elsewhere [19].

### 2.5. Statistical Analysis

ANOVA followed Dunnett's test was used for multiple comparisons. Two groups were compared using two-tailed unpaired Student's test. Correlations were analyzed using the Pearson's test. A *P* value <0.05 was considered statistically significant. Table and bar graph results are shown as mean ± standard error of the mean (SEM). 

## 3. Results

Presence of NeuGc-GM3 ganglioside was evident in 23 of 27 cases (85%) of neuroectodermal tumors (Table 1), as detected by immunohistochemistry using the specific monoclonal antibody 14F7. All cases of ESFT were positive, whereas some negative cases occurred in both NMYC-amplified and -nonamplified neuroblastoma. Absence of NeuGc-GM3 expression was not associated with any particular tumor site or with the use of preoperative polychemotherapy in high-risk patients.

**Figure 1:**
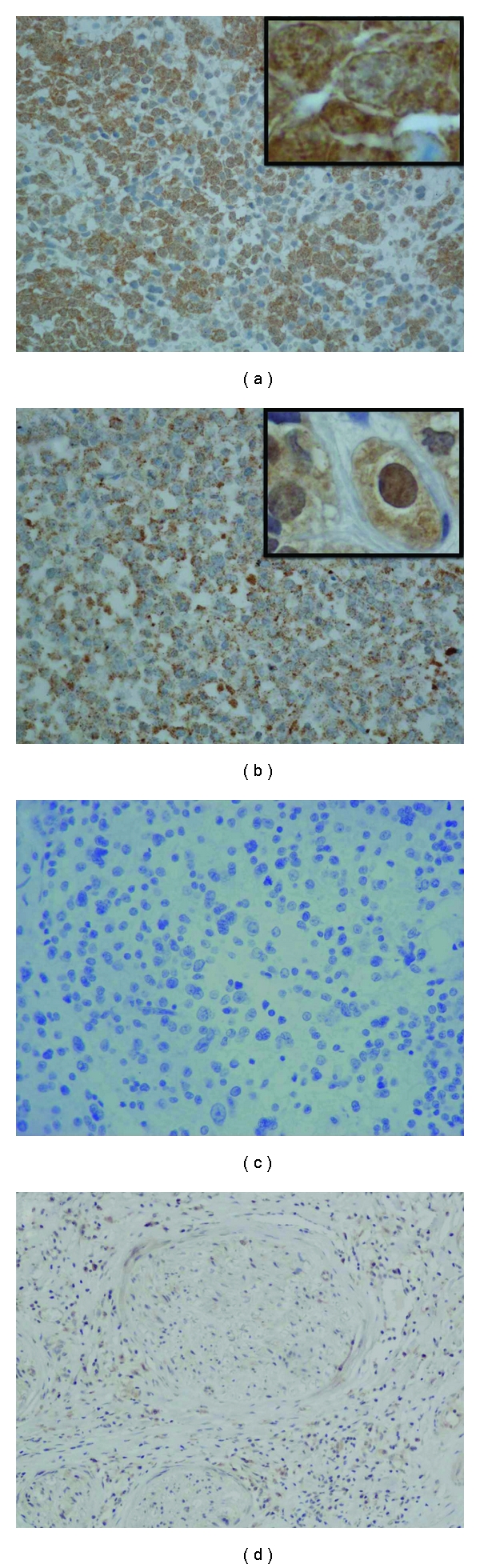
Immunohistochemical detection of NeuGc-GM3 ganglioside in neuroectodermal tumors. (a) Neuroblastoma (NMYC-amplified). (b) Ewing sarcoma family of tumors (ESFT). (c) Negative isotype control staining (mouse IgG1) in neuroblastoma. (d) No expression in nontumoral neural tissue. Original magnification 400X (a, b and c), 100X (d), 1,000X (insets).

In average, about 70% of tumor cells were positive for NeuGc-GM3 (see also Table 1). Immunoreactivity was moderate to intense in most tumors, showing a diffuse cytoplasmic and membranous staining in neuroblastoma (Figure 1(a)), with occasional nuclear positivity as previously reported for lung tumors [20]. A fine granular cytoplasmic pattern was detected in cases of ESFT (Figure 1(b)). Interestingly, most samples of neuroblastoma with an intense NeuGc-GM3 staining corresponded to patients with an age of less than 24 months. Adjacent adrenal tissue was positive for NeuGc-GM3 in the cytoplasmic compartment in some neuroblastoma cases analyzed, suggesting shedding of gangliosides from cancer cells, as described in renal tumors [9]. No expression of NeuGc-GM3 was detected in other nontumoral tissue (Figure 1(d)).

**Figure 2:**
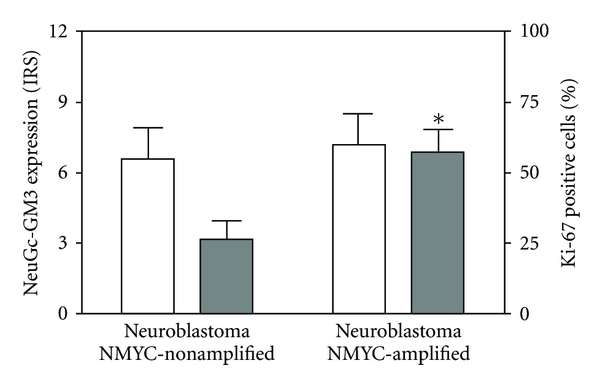
Expression of NeuGc-GM3 ganglioside and Ki-67 protein in NMYC-amplified and -nonamplified neuroblastoma. NeuGc-GM3 (open bars) was assessed with the immunoreactive score (IRS) and the percent of Ki-67 positive cells (closed bars) was used as a proliferation index. Data represent mean ± SEM. *P < 0.02 (*t* test)

All ESFT cases analyzed were positive for the *t*(11;22) translocation by FISH. No significant differences (*P* > 0.05) in NeuGc-GM3 expression were observed between NMYC-amplified and -nonamplified neuroblastoma, as assessed by the IRS (Figure 2). In the same line, no significant correlation was found between the percentage of cells positive for the Ki-67 proliferating antigen and the NeuGc-GM3 IRS (*P* > 0.05; *r* = 0.1638). As expected, tumors with NMYC amplification demonstrated a significantly higher expression (*P* < 0.02) of Ki-67 (see also Figure 2). Deletion of 1p36 was also confirmed in association with NMYC amplification in these cases. Taken together, the present data suggest that expression of NeuGc-GM3 is preserved in more aggressive neuroectodermal cancers.

## 4. Discussion

To the best of our knowledge, this is the first report on the expression of N-glycolylated gangliosides in pediatric neuroectodermal tumors. Our immunohistochemical study using a specific monoclonal antibody evidences NeuGc-GM3 expression in 85% of cases of neuroblastoma and ESFT. It is known that complex glycosphingolipids are abundant in cells of neuroectodermal origin [21], as well as in some epithelial cells [22]. Mammalian cells are covered by a dense glycocalyx, composed of glycolipids, glycoproteins, glycophospholipid anchors, and proteoglycans. Sialic acids attached to cell surface glycoconjugates play important roles in many physiological and pathological processes, including microbe binding that leads to infections, regulation of the immune response, and progression and spread of human malignancies [23]. The possibility that NeuGc-containing glycoconjugates are taken up directly from diet must be taken into account. However, the potential role of alternative biosynthetic pathways of NeuGc in human neoplasia, including pediatric tumors, is not known [24].

 The most common sialic acids in mammals are N-acetyl (NeuAc) and NeuGc neuraminic acids. The key step in the biosynthesis of NeuGc is the conversion of NeuAc to NeuGc, which is catalyzed by the cytidine monophospho-N-acetylneuraminic acid hydroxylase [25]. NeuGc-containing gangliosides are normal components of cell membranes in all mammals except human beings. The lack of expression of NeuGc in human tissues is due to inactivation by a deletion of the hydrolase gene [26]. However, neosynthesis of carbohydrate determinants and expression of NeuGc gangliosides were observed in human cancer, possibly by diet incorporation of nonhuman sialic acid from milk or meat [10]. NeuGc-GM3 has been detected in prevalent adult cancers such as nonsmall cell lung cancer [20], breast carcinoma [27], and melanoma [28]. 

Ganglioside expression in ESFT has received little attention in the literature. The expression of GD2 has been reported [29] but, to our knowledge, they have not been widely used for immunotherapy [30]. Our preliminary results may be used as background for potential developments in this area. Conversely, gangliosides have been extensively studied in neuroblastoma, and a complex expression profile showing variations between neuroblastoma tumors with different malignant potential was described [31]. Moreover, patterns of ganglioside expression were used as indicators to predict patient outcome as a prognostic indicator [31].

The overexpression of GD2 has been widely reported in neuroblastoma. It is expressed in virtually all cases, and it has been used as a target for immunotherapy after the development of anti-GD2 specific antibodies. The use of anti-GD2 murine or humanized antibodies for passive immunotherapy has shown to be an effective treatment of MRD, as reported by randomized studies [6]. However, this treatment requires frequent intravenous injections, and it may be associated to severe toxicity such as hypersensitivity reactions and capillary leak syndrome appearing in up to 25% of the cases. In addition, this treatment is only available for use within clinical trials in Europe and the USA, so it is currently not an option in less developed countries. In these settings, current therapies with high dose chemotherapy and autologous stem cell rescue are available, but novel treatments for MRD are needed to improve outcome.

Based upon our present results, active immunotherapy with the anti-idiotype vaccine racotumomab, targeting NeuGc-containing gangliosides, may be feasible in children with high-risk neuroblastoma. The wide expression of NeuGc-GM3 and the favorable toxicity profile of racotumomab [11–14] may make it an attractive option for clinical use. Our results were used as background for launching a Phase I evaluation of racotumomab in children with neuroectodermal tumors in our hospital. However, these results should be considered preliminary since a more detailed study on the expression of NeuGc-GM3 in different subsets of neuroblastoma is necessary. In addition, since expression of this gangliosides by neuroblastoma cells may be related to dietary uptake, different expression patterns in infants may be evident.

In our series, the use of preoperative chemotherapy may have changed the histopathological appearance and immunoreactivity. For instance, tumor cell subpopulations expressing NeuGc-GM3 may not be easily detectable in necrotic tumors after chemotherapy. As hypoxia-resistant cancer cells are known to have diminished response to chemotherapy, it is important to find potential target molecules for novel antitumor strategies [32]. In this context, resistant cancer cells could overexpress NeuGc-containing gangliosides under hypoxic conditions [33], but our study was not designed to assess this phenomenon. The ability of young children affected with neuroblastoma to develop an effective immune response to racotumomab vaccination may be limited, and this is also a focus of our current research.

In contrast to GD2 that is normally expressed in neural tissue of young children, NeuGc-containing gangliosides, including NeuGc-GM3, are virtually absent in normal human tissues, making these gangliosides immunogenic. In fact, antibodies that recognize NeuGc residues appear after administration of animal serum to humans [34]. Therefore, NeuGc-targeted immunotherapy may be considered an interesting candidate in neuroblastoma or other pediatric tumors such as ESFT. Although the number of cases is small, the present characterization of a specific neoantigen in neuroectodermal tumors may be of value for the design of immunotherapeutic protocols.

## Figures and Tables

**Table 1:** NeuGc-GM3 immunopositivity in neuroectodermal pediatric tumors.

Tumor variant	NeuGc-GM3		
	Positive cases^a^ (%)	Positive tumor cells^b^ (%)	Predominant intensity^c^
Neuroblastoma, NMYC-amplified	9/11 (81)	66 ± 11.6	2+/3+
Neuroblastoma, NMYC-nonamplified	9/11 (81)	69 ± 10.6	2+/3+
Ewing sarcoma family of tumors (ESFT)	5/5 (100)	71 ± 6.0	2+
Total	23/27 (85)	68 ± 6.3	2+

^a^Positive/total cases.

^b^Values are means ± SEM.

^c^Intensity of the positive staining was graded as 1+ = mild; 2+ = moderate; 3+ = intense.
